# Construction and Application of the Talent Training System in Colleges and Universities Based on the Fuzzy Analytic Hierarchy Process

**DOI:** 10.1155/2022/7295875

**Published:** 2022-09-14

**Authors:** Yan Yu, Jun Qiu

**Affiliations:** ^1^School of Economics and Management, Chong Qing Industry Polytechnic College, Chongqing 401120, China; ^2^School Work Department QuZhou College of Technology, Quzhou 324000, China

## Abstract

At present, the competition among talents to seize jobs is becoming more and fiercer. How to stand out in the fierce competition for talents and seize market resources is a problem that every graduate must think deeply. Domestic research on the composition of the implementation elements of talent training in colleges and universities from the perspective of excellent school talent training has achieved fruitful results. Their measures and implementation ideas mainly include the following aspects: first, analyze the system of talent training in colleges and universities; second, analyze the teaching methods of college teachers; third, analyze the future development direction of talents. Taking a university as a pilot unit, this paper constructs a talent training system based on the combination method of fuzzy hierarchy, determines the training goal according to students' own ability, formulates the talent training scheme according to the goal, determines the rectangular array relationship between the training system and students' own ability requirements and attaches importance to practical teaching. The algorithm used in this paper firstly establishes a hierarchical structure model and then combines the relevant measures of AHP to sort the weights of indicators and finally calculates the entropy value by quoting entropy weight. We use the fuzzy method combined with hierarchical analysis to evaluate the five indexes of students' own influencing factors, such as learning attitude, basic knowledge, cooperation ability, development ability, and professional ability. It can be seen that the algorithm model is more accurate and the error value is the smallest. Compared with their combination method, the fuzzy evaluation method and the hierarchical analysis method are practical, and the fuzzy method combined with the hierarchical analysis combination method has more practical significance. Finally, the employment situation of graduates is compared and analyzed to further highlight the effectiveness of the model.

## 1. Introduction

Fuzzy map is generated by fuzzy membership function of each parameter in a suitable karst area, and the weight of each layer is assigned by the AHP method, and finally the final fuzzy-AHP map is generated [1]. For the popular Novel Coronavirus, fuzzy AHP is used to determine the weight of the existing criteria, and fuzzy TOPSIS is used to determine the safest area, that is ready to implement the new normal [2]. The competitive technology between China and Africa is transferred at any time, and the method in this paper is used to calculate the weight [3]. An appropriate proposed framework was implemented using the fuzzy analytic hierarchy process in a palm oil industry in Indonesia to confirm its applicability and usefulness. Research shows that the environmental dimension is the most sustainable supplier standard, followed by the economic and social dimensions [4]. Taking Herat, Afghanistan as an example, the suitability of the GIS wind farm is evaluated by the fuzzy AHP multi-criteria method [5]. We combine the fuzzy analytic hierarchy process with the fuzzy mixed multi-criteria method and study the most influential and conflicting criteria in economy, service level, environment, society, and risk [6]. Our evaluation model of financial audit research in the financial shared service mode is established by the fuzzy method combined with the hierarchical analysis combination method and order estimation methodology through similarity of solutions [7]. We use the fuzzy method and the hierarchical analysis combination method to find the optimal system of processing cigarette manufacturing [8]. We use the fuzzy method and the hierarchical analysis combination method to calculate the relative weights of relevant criteria. Finally, based on these weights, we use the ranking technology approaching the ideal solution to rank banks, and the results show that the major banks rank in the top three, respectively [9]. In the Craig–Harris method to measure the productivity level of farms, a fishbone diagram is used to analyze the reasons for productivity decline, and the fuzzy method combined with the class analysis combination method is proposed to improve productivity, which is based on the Craig–Harris method to measure productivity [10]. Researchers have developed and applied various standards and methods to find suitable rainwater harvesting sites and technologies. The main goal of the work is to use the fuzzy method combined with the hierarchical analysis combination method to assign weights to various standards involved in selecting suitable RWH sites in the Kandi subdivision of Murshidabad District, India [11]. Spectrum seems to be the lifeblood of wireless communication, and the fuzzy analytic hierarchy process seems to be an appropriate solution for spectrum allocation among SUs without interference between themselves [12]. Double-skin sickle (DSF) has been paid more attention by manufacturers in recent years because of its practical and aesthetic characteristics. In the previous research, our research group used the AHP method to evaluate DSF [13]. The fuzzy method combined with the hierarchical analysis and combination method is used to evaluate the quality of online commercial services, and the research results can be used by other e-commerce enterprises to improve the service quality according to the standards and sub-standards that experts think are important [14]. The supply chain adopted in medicine and medicine requires an orderly implementation plan for each strategic element of the pharmaceutical supply chain and uses the fuzzy method combined with the hierarchical analysis and combination method to minimize its weight on cost and time, so that decision makers can optimize the economy of PSC and create space [15]. In this paper, an extended FAHP model is proposed, in which two fuzzy comparison rectangular matrices are represented by a special class of fuzzy numbers. Then it is proved that a large number of FAHP methods can be simply written into the proposed E-FAHP structure [16]. We use the fuzzy method combined with hierarchical analysis to improve the understanding of the determinants of effective hotel websites and provide practical suggestions in order to formulate appropriate strategies to transform website visitors into customers [17]. In various cases of decision analysis, we adopt two popular methods: analytic hierarchy process (AHP) and fuzzy AHP. Both methods deal with random data, and the decision results can be determined by some criterion decision processes [18]. The purpose of this study is to assist the catering industry and credit card issuing banks to establish performance evaluation indicators of marketing alliance by means of expert Delphi method, fuzzy method combined with hierarchical analysis, and balanced scorecard [19]. Fuzzy analytic hierarchy process (FAHP) is used to determine the weight of each sub-criterion according to the evaluation of experts. When there is no pairwise comparison of existing alternatives, we adjust to use sustainable development [20]. Fuzzy method combined with hierarchical analysis and ARAS-G is used to evaluate the performance of logistics in OECD countries [21]. Application of the fuzzy analytic hierarchy process and the TOPSIS method in cross-domain collaborative recommendation with fuzzy visual representation [22]. Researchers use the fuzzy method combined with hierarchical analysis to analyze the effective judgment of trademark infringement compensation cases in Beijing Intellectual Property Court in 2018 [23]. In the case study of an Indonesian sole manufacturer seeking to expand its business to the international market, the weights of the fuzzy method combined with the hierarchical analysis and combination method are adopted, and PROMETHEE is used to sort the schemes [24]. Fuzzy AHP and completely consistent implementation are used to rank the relative importance of these CSFs and their dimensions for continuous academic quality assurance and ABET certification [25].

## 2. The Necessity of Personnel Training in Undergraduate Colleges and Universities

### 2.1. Connotation of Personnel Training

To construct the talent training mode system in colleges and universities, the first point is to determine what system should be adopted to train talents. That is, through what kind of thinking mode and practical means to cultivate talents who conform to the development of the times. Therefore, to cultivate talents, we must first master the correct talent training system, so as to realize the terminal of talent training in the true sense for people's needs. The way of talent training is the professional knowledge, learning ability, future trend, and the way to achieve the ideal goal determined by the school for students. It collects and determines the characteristics that talents should have and also effectively embodies the educational philosophy and practice. Choose the appropriate way to realize the talent training mode and reference blueprint and cultivate talents with certain innovative ability. We can neither be subject to the traditional theoretical classroom teaching experience, blindly go with the flow, nor reform the training system that is arbitrarily enlarged to the whole school. Similarly, as the standard style of personnel training, it is formed by the integration and adjustment of many scientific theories, practical operations, and other factors, and many influencing factors together form a complex entity, which not only includes the theoretical chapters of personnel training but also reflects the reality of traditional educational practice training talents in recent years. This is a compound combination formed by the mutual influence and combination of many different links, and their multiple ideas together constitute the connotation of the current standardized talent training mode.

### 2.2. Attach Importance to Vocational Education

The implementation elements of vocational personnel training should pay attention to the cultivation of students' double-base ability. While improving vocational ability, we should uphold the sustainable development concept of lifelong learning and establish a training system in the field of vocational education that runs through from top to bottom and connects from left to right. Pave a smooth road for continuously improving professional quality and learning and development ability. This step is often realized through the school cultural education system. The school provides a basic place for students to cultivate and improve their professional ability. Only by receiving a certain period of school culture education and acquiring basic professional knowledge in school to meet the graduation requirements can students realize their personal value and further enhance their professional ability when they really go to work in the future. Vocational education is dominated by technical and professional characteristics, upholds the basic concepts of employment orientation, quality-oriented, unity of knowledge and practice, and lifelong development and cultivates students' vocational ability to meet the needs of social development.

### 2.3. Education around Students

Around the mode of students' education, the main object of talent education is the students who have received ordinary school education and have a certain knowledge base, and the training of vocational education talents at an undergraduate level is no exception. Students who graduated from ordinary high schools are also the main body of talent education. Therefore, we must adhere to the basic principle of “taking students as the main body and teachers as the leading factor”. Talent education focuses on the basic teaching principles of the students' teaching mode, in order to explore the development laws of nature and society and constantly learn and pursue progressive learning. When formulating the implementation elements of personnel training, colleges and universities must pay attention to taking students as the main body, carry out education around students and accurately understand the training information fed back by students through the evaluation of teaching methods and students' questionnaire survey, which can also make schools clearly recognize their own shortcomings and correct them. We should not only take this measure but also formulate a personalized training mode for students, carefully understand the real needs of students and give full play to the leading role that school education can play in the process of personnel training. After the talent training mode has been tested in practice, we can get feedback by investigating the previous students' evaluation of the implementation of teaching content and the employment situation after graduation, so that the training mode can be continuously improved and the talent training mode can be continuously reformed. Realize the trinity consultation mechanism among schools, enterprises, and students. Both sides understand each other's pursuit of interests, so that the training of talents in schools is more realistic, and the implementation elements of personnel training tend to be more ideal.

## 3. Fuzzy Analytic Hierarchy Process Algorithm

### 3.1. Building a Hierarchical Model

According to the analysis of their own needs, the goal, key impact factors, and program objects can be divided into goal level, method level, and program level according to their connection relationship, and the corresponding hierarchical structure diagram is constructed to form a hierarchical structure model.

#### 3.1.1. Constructing Judgment Matrices against Each Other

According to the weights of the two factors, a judgment matrix is formed:

(1) $$\begin{array}{c} Judgment\;matrix=\left\{\begin{array}{c} a_{11},a_{12},\ldots ,a_{1\left(n-1\right)},a_{1n}\\ a_{21},a_{22},\ldots ,a_{2\left(n-1\right)},a_{2n}\\ \ldots ,\ldots ,\ldots ,\ldots ,\ldots ,\ldots \text{,}\\ a_{n1},a_{n2},\ldots ,a_{n\left(n-1\right)},a_{n\left(n-1\right)},a_{nn} \end{array}\right\}\text{.} \end{array}$$

#### 3.1.2. Single Layer Weight Calculation

Multiply the factors of each row of matrix *A* to calculate the product as

(2) $$\begin{array}{c} M_{i}=a_{i1}*a_{i2}*\ldots *a_{i\left(n-1\right)}a_{in}\text{.} \end{array}$$

Calculate the root of *N* to the nth power, and the expression is as follows:

(3) $$\begin{array}{c} \bar{V}=\left[\sqrt[{n}]{N_{1}},\sqrt[{n}]{N_{2}},\ldots ,\sqrt[{n}]{N_{\left(n-1\right)}},\sqrt[{n}]{N_{n}}\right]\text{.} \end{array}$$

The weight vector of each factor is obtained by processing vector $\bar{V}$ with the following normalization formula:

(4) $$\begin{array}{c} V_{i}=\frac{\bar{V_{i}}}{\sum_{i=1}^{n}\bar{V_{i}}}\text{.} \end{array}$$

#### 3.1.3. Consistency Test of Matrix

Calculate the maximum eigenvalue of matrix *A* as

(5) $$\begin{array}{c} \lambda_{\max}=\frac{1}{\sum_{i=1}^{n}{\left(AV\right)}_{i}/V_{i}},\\ {\left(AV\right)}_{i}=\left(V_{1},V_{2},V_{3},V_{4}\right)\left\{\begin{array}{c} A_{11},A_{12},A_{13},A_{14}\\ A_{21},A_{22},A_{23},A_{24}\\ A_{31},A_{32},A_{33},A_{34}\\ A_{41},A_{42},A_{43},A_{44} \end{array}\right\}\text{.} \end{array}$$

Expressions for calculating consistency metrics is

(6) $$\begin{array}{c} C.I=\frac{\lambda_{\max}-n}{n-1}\text{.} \end{array}$$

According to the judgment matrices of different orders, the corresponding average random consistency index R.I. can be obtained by looking up the table, and finally the consistency ratio can be calculated by using it.

#### 3.1.4. Total Sort of All Hierarchies

After sorting the importance and weight of the above single-level factors, all levels will be sorted in this way. The calculation method is as follows:

(7) $$\begin{array}{c} V_{i}^{\left(k\right)}=\overset{m}{\underset{j=1}{\sum }}p_{ij}^{\left(k\right)}{wj}^{\left(k-1\right)}i=1,2,3,..,n\text{.} \end{array}$$

In the same way, the consistency test of the total ranking is also carried out.

### 3.2. Fuzzy Analytic Hierarchy Process

The phase test of fuzzy matrix is established, and a matrix is defined as

(8) $$\begin{array}{c} R={\left(r_{ij}\right)}_{n\times n}\text{.} \end{array}$$

The fuzzy complementary matrix of the matrix can be obtained by satisfying the following conditions:

If the formula satisfies:0 ≤ *r*_*ij*_ ≤ 1, (*i*=1,2, *n*; *j*=1,2, *n*), then fuzzy array *R* is a fuzzy complementary matrix.

The fuzzy consistent matrix can be obtained by satisfying the following conditions:

(9) $$\begin{array}{c} r_{ij}=r_{iK}-r_{jk}+0.5\text{.} \end{array}$$

### 3.3. Index Scoring Weight Setting

**Figure 1:**
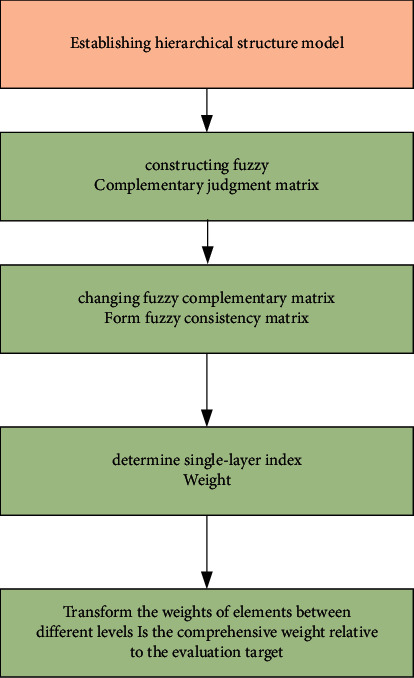
Weight setting steps of the bidding score.

We use the fuzzy analytic hierarchy process to construct all indicators and calculate the basic steps of scoring weights as shown in Figure 1.

Construct fuzzy complementary judgment matrix, compare the importance of different influencing factors in pairs and transform it into fuzzy complementary judgment matrix as

(10) $$\begin{array}{c} R=\left\{\begin{array}{c} R_{11},R_{12},R_{13},R_{14}\\ R_{21},R_{22},R_{23},R_{24}\\ R_{31},R_{32},R_{33},R_{34}\\ R_{41},R_{42},R_{43},R_{44} \end{array}\right\}\text{.} \end{array}$$

The fuzzy complementary matrix is summed by rows, and the expression is

(11) $$\begin{array}{c} r_{i}=\sum_{j=1}^{n}r_{ij},i=1,2,..,n\text{.} \end{array}$$

Carry out mathematical transformation into the following formula:

(12) $$\begin{array}{c} r_{ij}=\frac{\left(r_{i}-r_{j}\right)}{2n+0.5}\text{.} \end{array}$$

For the neat fuzzy consistency matrix, the factor of each row of the matrix is multiplied by the power product method:

(13) $$\begin{array}{c} R_{i}=r_{i1}\times r_{i2}\times \ldots \times r_{i\left(n-1\right)}\times r_{in}\text{.} \end{array}$$

Calculate the nth root of *R*_*i*_ as

(14) $$\begin{array}{c} \bar{R}=\left[\sqrt[{n}]{R_{1}},\sqrt[{n}]{R_{2}},\ldots ,\sqrt[{n}]{R_{n-1}},\sqrt[{n}]{R_{n}}\right]\text{.} \end{array}$$

Normalizing the above vectors as

(15) $$\begin{array}{c} R_{i}=\frac{\bar{R_{i}}}{\sum_{i=1}^{n}\bar{R_{i}}}\text{.} \end{array}$$

Ranking vectors of fuzzy complementary judgment matrices as

(16) $$\begin{array}{c} w={\left(w_{1},w_{2},..,w_{n}\right)}^{T}\text{.} \end{array}$$

Its constraints are as follows:

(17) $$\begin{array}{c} w_{i}=\frac{\sum_{j=1}^{n}r_{ij}+n/2-1}{n\left(n-1\right)}\;i=1,2,..,n\text{.} \end{array}$$

Calculate the compatibility index of fuzzy judgment matrix: as

(18) $$\begin{array}{c} I\left(A,R\right)=1/n^{2}\overset{n}{\underset{i=1}{\sum }}\overset{n}{\underset{j=1}{\sum }}\left|r_{ij}-p_{ij}\right|\text{.} \end{array}$$

### 3.4. Triangular Fuzzy Number

The relative importance of the two influencing factors is calculated. If*W*_1_, *W*_2_are triangular fuzzy numbers, the probability degree of (*W*_1_ ≥ *W*_2_)is defined as *V*(*W*_1_ ≥ *W*_2_).

When

(19) $$\begin{array}{c} m_{1}\geq m_{2},V\left(W_{1}\geq W_{2}\right)=1\text{.} \end{array}$$

When

(20) $$\begin{array}{c} m_{1}\leq m_{2},V\left(W_{1}\geq W_{2}\right)=\left\{\begin{array}{c} \frac{l_{2}-u_{1}}{\left.\left(m_{1}-u_{1}\right)-\left(m_{2}-l_{2}\right)\right)},l_{2}\leq u_{1},\\ 0,l_{2}>u_{1}\text{.} \end{array}\right. \end{array}$$

The probability that the triangular fuzzy number W is greater than *k* triangular fuzzy numbers *W*_1_ is defined as

(21) $$\begin{array}{c} V\left(W\geq {\;W}_{1}\;,{\;W}_{2},..,{\;W}_{k}\;\right)=minV\left(W\geq {\;W}_{1}\right)\left(i=1,2,..,n\right)\text{.} \end{array}$$

In order to introduce entropy weight into the model, several definitions are introduced below.

Set projection of all indicators as

(22) $$\begin{array}{c} P_{ij}=\frac{x_{ij}}{\overset{m}{\underset{i=1}{\sum }}x_{ij}}\text{.} \end{array}$$

According to the information conclusion, the entropy value output by each index *i* is

(23) $$\begin{array}{c} E_{i}=k\overset{m}{\underset{i=1}{\sum }}P_{ij}\ln\left(P_{ij}\right),i=1,2,..,n,\\ k={\left(\ln\;m\right)}^{-1}\text{.} \end{array}$$

Deviation is

(24) $$\begin{array}{c} d_{i}=1-E_{i}\left(i=1,2,\ldots ,n\right)\text{.} \end{array}$$

## 4. Experimental Study

### 4.1. Basic Information of Research

In this experiment, the fuzzy analytic hierarchy process is used to investigate college students at different levels to better build a talent system in colleges and universities, involving basic information such as students' age, gender, grade, interest field, and school. The subjects of this survey are 200 students from 20 universities in China, and their interest research fields are mainly concentrated in 5 fields. The investigation status are shown in Table 1.

Among the 200 respondents, we can see that the proportion of students majoring in scientific research is the least, and students are more inclined to study big data applications and financial fields. According to the fuzzy analytic hierarchy process studied in this paper, we carry out feedback analysis and statistics of the existing personnel training situation.

**Figure 2:**
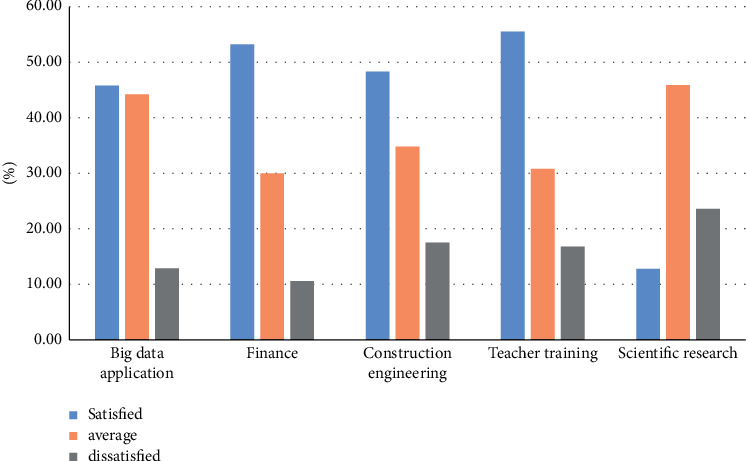
Students' satisfaction with the training system of colleges and universities.

Whether the respondents in the five research fields are satisfied with the training system used by talents in schools today, the survey results are shown in Figure 2.

**Figure 3:**
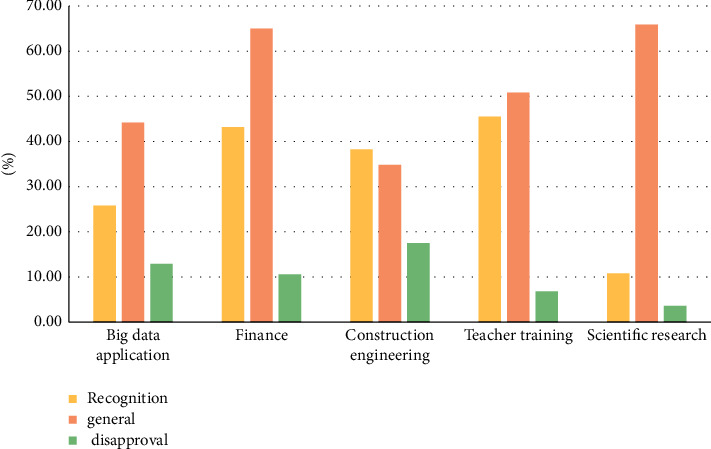
Students' recognition of teaching methods in colleges and universities.

Continue to investigate whether the respondents in the five types of research fields recognize the teaching methods in colleges and universities today. The survey results are shown in Figure 3.

**Figure 4:**
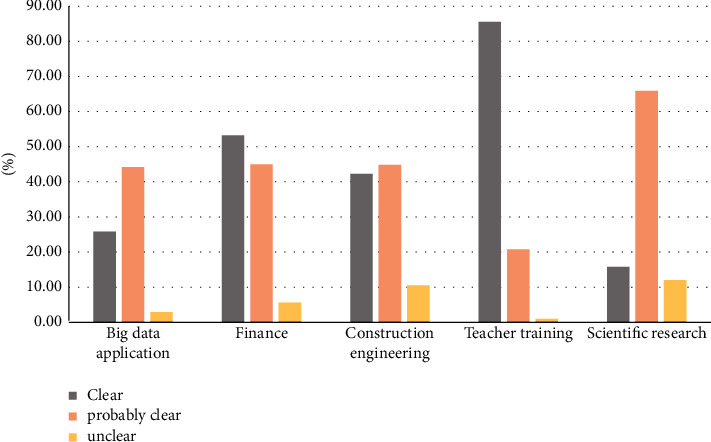
Students' clarity on the future development direction.

Finally, whether the respondents in the five research fields have a clear understanding of the future employment direction, the survey results are shown in Figure 4.

### 4.2. Model Comparison

In order to highlight the advantages of the fuzzy method combined with hierarchical analysis in the talent training system of colleges and universities, this paper studies and analyzes the students' self-influence indicators in the talent training system of five research fields together with the fuzzy average algorithm and the analytic hierarchy process.

(25) $$\begin{array}{c} MAE=\frac{1}{K}\overset{K}{\underset{K=1}{\sum }}\left|{\hat{y}}^{k}-y^{\left(k\right)}\right|,\\ RMSE=\sqrt{\frac{\sum_{K=1}^{K}{\left({\hat{y}}^{k}-y^{\left(k\right)}\right)}^{2}}{K}},\\ R^{2}=\frac{{\left[\sum_{K=1}^{K}\left(y^{k}-{\hat{y}}_{Ave}\right)\left(y^{\left(k\right)}-{\hat{y}}_{Ave}\right)\right]}^{2}}{\sum_{K=1}^{K}\left({\hat{y}}^{k}-{\hat{y}}_{Ave}\right)\sum_{K=1}^{K}\left(y^{\left(k\right)}-{\hat{y}}_{Ave}\right)}\text{.} \end{array}$$

Fuzzy method combined with the class analysis method to evaluate students' self-learning attitude, basic knowledge, cooperation ability, development ability, and professional ability. The research data are shown in Table 2.

**Figure 5:**
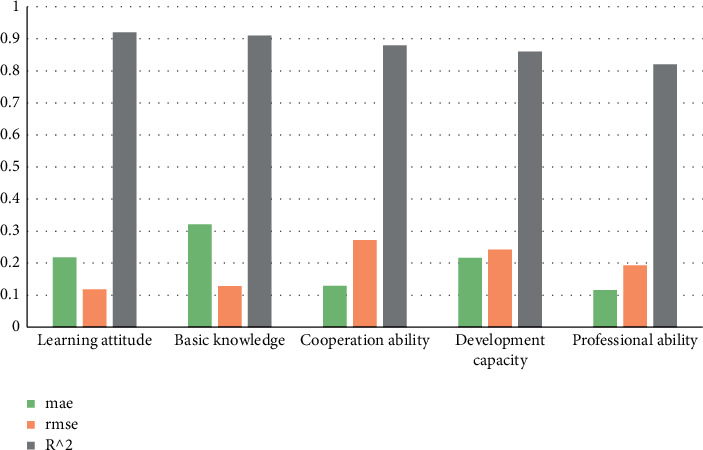
Performance analysis of the model on its own indicators.

Carry out histogram statistics on the data in the above table as shown in Figure 5.

Fuzzy evaluation method evaluates students' self-learning attitude, basic knowledge, cooperation ability, development ability, and professional ability. The research data are shown in Table 3.

**Figure 6:**
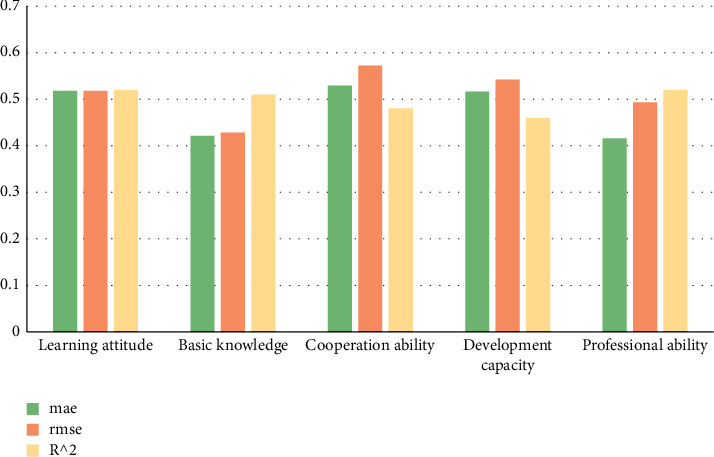
Performance analysis of the model on its own indicators.

Carry out histogram statistics on the data in the above table as shown in Figure 6.

Analytic hierarchy process (AHP) evaluates students' learning attitude, basic knowledge, cooperation ability, development ability, and professional ability. The research data are shown in Table 4.

**Figure 7:**
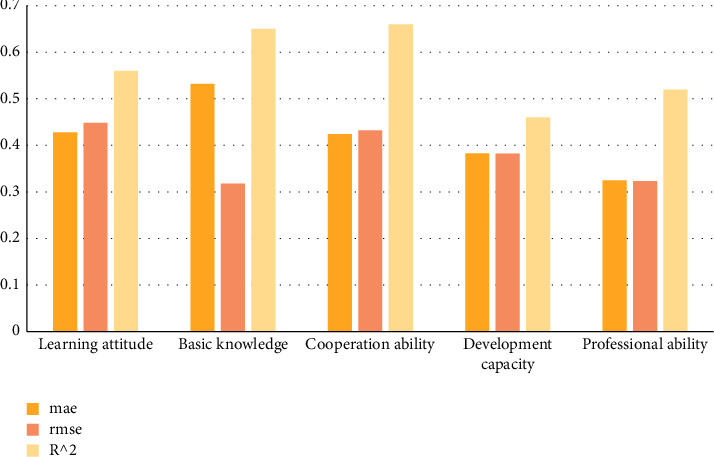
Performance analysis of the model on its own indicators.

Carry out histogram statistics on the data in the above table as shown in Figure 7.

### 4.3. Contrast Experiment

**Figure 8:**
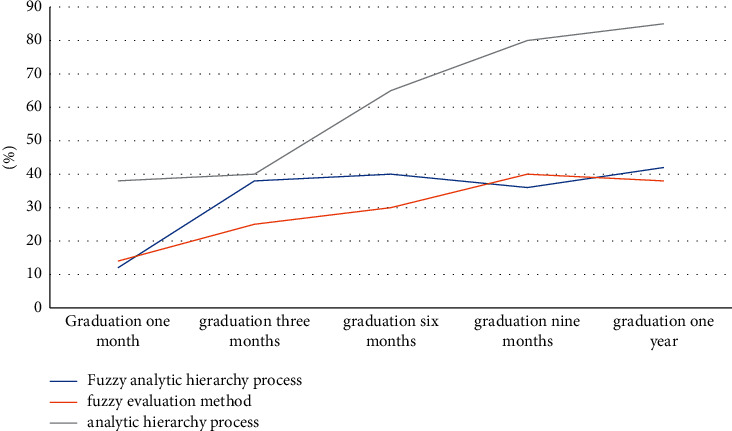
Employment situation under the talent training system of three models.

In view of the construction of the talent training system, this paper investigates the students in 5 fields in 20 universities, and use the fuzzy method combined with the hierarchy analysis combination method to construct and apply it. Finally, show whether the model is effective according to their employment situation and compare and analyze the fuzzy evaluation method and the analytic hierarchy process of constructing the talent training system with their combination method, as shown in Figure 8:

## 5. Conclusion

The construction of talent education and training system in colleges and universities needs a first-class education system with perfect functions, efficient operation, excellent service, and strong guarantee. Among them, it needs the joint efforts of teachers and students to become the winner in the talent market. Based on the fuzzy method combined with the class analysis and combination method, this paper has the following research results:

1. The proportion of students involved in scientific research is the least. We should encourage students to conduct scientific research and strengthen the motherland with the power of science.
2. Students in various research fields have low recognition of teachers' teaching methods, and teachers can change their inherent teaching thinking and mode in accordance with their aptitude.
3. Fuzzy analytic hierarchy process combined method is superior to the single fuzzy evaluation method and the analytic hierarchy process, and its root mean square error is the smallest, and its determination coefficient is closest to 1.
4. In the comparative experiment, the employment situation of students after graduation is used to test whether the talent training system in colleges and universities is effective, and the method advocated in this paper is superior to the other two methods.

## Figures and Tables

**Table 1:** Basic information of respondents.

Category	Investigation items	Percentage proportion
Gender	Men	48%
	Women	52%
	Senior year	

Grade	Ken-ichi	20%
	Yanji	21%
	Yan san	25%
		34%

Areas of interest	Big data application	32%
	Finance	24%
	Construction engineering	20.8%
	Teacher training	20%
	Scientific research	3.2%

**Table 2:** Data of error, root mean square error, and determination coefficient of indicators.

Self-influence index	mae	rmse	*R* ^2^
Learning attitude	0.218	0.118	0.92
Basic knowledge	0.321	0.128	0.91
Cooperation ability	0.129	0.272	0.88
Development capacity	0.216	0.242	0.86
Professional ability	0.116	0.193	0.82

**Table 3:** Data of error, root mean square error and determination coefficient of indicators.

Self-influence index	mae	rmse	*R* ^2^
Learning attitude	0.518	0.518	0.52
Basic knowledge	0.421	0.428	0.51
Cooperation ability	0.529	0.572	0.48
Development capacity	0.516	0.542	0.46
Professional ability	0.416	0.493	0.52

**Table 4:** Data of error, root mean square error, and determination coefficient of indicators.

Self-influence index	mae	rmse	*R* ^2^
Learning attitude	0.428	0.448	0.56
Basic knowledge	0.532	0.318	0.65
Cooperation ability	0.424	0.432	0.66
Development capacity	0.383	0.382	0.58
Professional ability	0.325	0.323	0.59

## Data Availability

The data used to support the findings of this study are available from the corresponding author upon request.
